# Association Between Smartphone Usage and Primary Headaches in Taif, Saudi Arabia: A Hospital-Based Study

**DOI:** 10.7759/cureus.53859

**Published:** 2024-02-08

**Authors:** Adnan A Mubaraki, Taif S Alharthi, Razan M Alkhoshi, Shahad A Alamri, Shahad K Alhunbusi, Raghad M Altwiraqi

**Affiliations:** 1 Department of Medicine, Faculty of Medicine, Taif University, Taif, SAU

**Keywords:** smartphone overuse, primary headache, migraine, electronic devices, addiction

## Abstract

Background

Integrating smartphones into human life has transformed various daily activities. Numerous symptoms, including headaches, have been linked to smartphone use. The excessive use of these devices raises significant health concerns. This study aimed to investigate the relationship between smartphone use and the progression, type, and severity of primary headaches, as well as the necessity for both abortive and prophylactic medications in treating such conditions.

Materials and methods

In this study, we utilized a cross-sectional survey involving 403 participants aged 14 years and older, all experiencing primary headaches and seeking care at three governmental hospitals in Taif, Saudi Arabia. The participants were divided into two groups based on their smartphone usage: high and low smartphone users. Data were collected through a hospital-based questionnaire administered across the three centers. Descriptive analysis and Pearson chi-square tests were conducted using IBM SPSS Statistics for Windows, Version 21 (released 2012; IBM Corp., Armonk, New York, United States).

Results

Of the participants, 128 (31.8%) were classified as low users, while 275 (68.2%) were identified as high users. The most frequently reported type of headache was undiagnosed headache, among 109 (27%), followed by migraine, at 86 (21.3%). Headaches were severe in 112 (40.9%) of cases and moderate in 134 (48.9%) of users. Around 62 (72.1%) of participants diagnosed with migraine reported a severe impact on their daily lives. In terms of medication usage, 166 (60.6%) of participants reported consuming zero to five pills monthly, while 52 (19%) reported taking more than 10 pills monthly. Additionally, 58 (21.2%) of participants utilized prophylactic medications.

Conclusion

No significant association was observed between smartphone use and the impact of headaches. Notably, pain severity was higher among low smartphone users who reported a high intake of medications. Migraine was the most severe and intense headache type. On average, the participants consumed fewer than five pills per month.

## Introduction

Smartphones have become an integral part of every community [1]. The ownership and usage of smartphones have exponentially increased, with over six billion people worldwide currently using smartphones; this number is expected to increase in the coming years [2]. Integrating smartphones into human life and society has reshaped daily work, education, and leisure activities [3]. Despite their numerous advantages, such as internet access, multimedia capabilities, navigation, and communication [4], the excessive use of these devices raises health concerns [5]. The symptoms experienced by patients vary depending on the frequency of smartphone usage throughout the day [6]. Excessive smartphone use has been associated with issues such as obesity, fatigue, difficulty concentrating, the development of musculoskeletal pain, and, most commonly, headaches [3]. Primary headaches are characterized by recurrent or persistent headaches without an apparent underlying cause, distinguishing them from secondary headaches that arise in specific triggering situations [7]. According to the International Classification of Headache Disorders (ICHD), migraine and tension-type headaches are the most prevalent types of primary headaches [8]. A prior study conducted in Saudi Arabia indicated that tension-type headaches are more prevalent than migraines. Generally, the prevalence of both types is higher in Saudi Arabia than the global average [9].

Various causative mechanisms, including electromagnetic fields, eye and ear strain, neck posture, reduced sleep, mental strain, and local thermal effects, contribute to the association between smartphone use and headaches [10]. A study conducted in Poland found that smartphone usage led to an increase in the permeability of the blood-brain barrier and altered the activities of certain opioid substances, both of which are linked to headaches [6].

Numerous previous studies have explored the correlation between smartphone use and headaches, examining the severity and types of headaches and the utilization of medications. In an observational study conducted in Pakistan between 2021 and 2022 among patients with migraines categorized as high and low smartphone users, the results indicated that excessive smartphone use could exacerbate pain intensity and diminish the effectiveness of treatments for patients with migraines [11].

A hospital-based cross-sectional study conducted in 2018 involving 400 participants aimed to investigate the relationship between smartphone use and headache characteristics in individuals with primary headaches. The findings indicated that patients with high smartphone use exhibited similar headache characteristics as non-smartphone users but with an elevated need for medication and less relief from symptoms in the high smartphone user group [10]. Similarly, a study conducted among university students in Turkey reported significantly higher levels of headache complaints among high smartphone users, with an observed increase in both the frequency and duration of headaches within this group [12].

## Materials and methods

Study design and population

A cross-sectional study was performed in three prominent governmental hospitals in Taif, Saudi Arabia, between March and October 2023. The participants included patients with primary headaches receiving follow-up care at these centers. Exclusion criteria involved participants aged less than 14 years, those with known secondary causes of headaches, or individuals who declined to participate.

Sample size and data collection tools

The Raosoft Sample Size Calculator was used to determine the sample size, considering the population size, a 95% confidence range, and a 5% margin of error [13]. The initial projected sample size was 400. Data collection was performed randomly using a hospital-based questionnaire containing multiple-choice questions distributed among participants in various centers across Taif. The questionnaire consisted of four parts and was administered by a designated data collector. An online informed consent process was implemented as a prerequisite for survey participation. Section one of the questionnaire focused on gathering sociodemographic information and basic academic details, including age, gender, education level, and employment status.

Section two evaluated smartphone addiction using the Smartphone Addiction Questionnaire (SAQ). After obtaining permission from the original publisher, a questionnaire consisting of 11 items based on the Diagnostic and Statistical Manual of Mental Disorders (DSM) V criteria for substance use disorders was prepared to score smartphone and internet overuse [10,14]. The maximum score for the questionnaire was 11. Each item carried a maximum score of one, with responses marked as zero for "no" and one for "yes." Participants scoring zero to one on the SAQ or using smartphones solely for calling were categorized as low smartphone users, while those with a score greater than one were classified as high smartphone users.

Section three encompassed details regarding the type and severity of headaches, assessed through the Visual Analogue Scale (VAS) [15]. The VAS is a self-reported measure that gauges pain severity by marking a point along a 10 cm line, ranging from "no pain" on the left end (0 cm) to "worst pain" on the right end (10 cm). Additionally, the Headache Impact Test (HIT-6) [16] was utilized to evaluate a broad spectrum of adverse impacts of headaches on social functioning, role functioning, vitality, cognitive functioning, and psychological distress. Patients responded to each item with one of five options: "never," "rarely," "sometimes," "very often," or "always," corresponding to scores of 6, 8, 10, 11, and 13, respectively. The sum of these responses ranged from 36 to 78, with a higher score indicating a more significant impact of headaches on the respondent's daily life. Section four focused on the treatment history for headaches.

Data analysis

The data were collected, examined, and input into IBM SPSS Statistics for Windows, Version 21 (released 2012; IBM Corp., Armonk, New York, United States). All statistical methods employed were two-tailed, with an alpha level of 0.05. Descriptive analysis entailed presenting the frequency distribution and percentage for study variables, encompassing participants' personal data, smartphone usage, headache information, and associated clinical data, including its impact. Cross-tabulation was conducted to illustrate all bivariate relationships, using the Pearson chi-square test for significance and an exact probability test in cases of small frequency distributions.

Ethical considerations

This study received approval from the Research Ethics Committee of Armed Forces Hospitals (Approval No. 2023-733) and the Directorate of Health Affairs, Taif Research and Studies Department (Approval No. 799). The participants were informed about the study objectives at the beginning of the survey, and their informed consent was subsequently obtained.

## Results

A total of 403 smartphone users were enrolled, with 128 (31.8%) categorized as low smartphone users and 275 (68.2%) identified as high users. In the 15-39 age group, 50% of individuals were classified as low smartphone users, contrasting with 66.9% among high users. Among those aged 60 or above, 10.9% fell into the low smartphone user category compared to 1.5% in the high smartphone user group, showing statistical significance (P =.001). Regarding education levels, 48.4% of low smartphone users had secondary education or below, while the corresponding figure for high smartphone users was 29.1%, with 67.3% being university graduates (P =.001). Additionally, 77.3% of low smartphone users were unemployed compared to 59.3% of high smartphone users (P =.001) (Table 1).

**Table 1:** Demographic characteristics among smartphone users according to level of use P: Pearson X2 test; $: exact probability test; * P < 0.05 (significant)

Demographic data	Total		Smartphone users				P-value
			Low smartphone users		High smartphone users		
	No	%	No	%	No	%	
Age in years							.001*
15-39	248	61.5%	64	50.0%	184	66.9%	
40-59	137	34.0%	50	39.1%	87	31.6%	
60+	18	4.5%	14	10.9%	4	1.5%	
Gender							.097
Male	93	23.1%	23	18.0%	70	25.5%	
Female	310	76.9%	105	82.0%	205	74.5%	
Educational level							.001*^$^
Secondary/below	142	35.2%	62	48.4%	80	29.1%	
University graduate	249	61.8%	64	50.0%	185	67.3%	
Post-graduate	12	3.0%	2	1.6%	10	3.6%	
Employment							.001*
Unemployed/retired	262	65.0%	99	77.3%	163	59.3%	
Employed	141	35.0%	29	22.7%	112	40.7%	

The prevalence of primary headaches among smartphone users in Taif, Saudi Arabia

Of the study participants, 274 (68%) reported experiencing primary headaches, while 129 (32%) did not. The predominant type of reported headache was an undiagnosed headache (27%; 24.2% among low users and 28.4% among high users), followed by migraines (21.3%; 25% and 19.6%, respectively) and non-specific headaches (9.4%; 12.5% and 8%, respectively), with recorded statistical significance (P =.025). Furthermore, 40.9% of users experienced severe headaches (50.5% and 35.8%, respectively), while 48.9% reported moderate headaches (37.9% and 54.7%, respectively; P =.028). Among the participants, 38% noted improved headache pain, while 14.6% reported worsened pain (8.4% and 17.9%, respectively). Among those with headaches, 60.6% took zero to five pills monthly (47.4% and 67.6%, respectively), and 19% consumed more than ten pills monthly (30.5% and 12.8%, respectively; P =.001). Additionally, 21.2% of headache sufferers used prophylactic medications (26.3% and 18.4%, respectively; P =.129). Approximately 34.7% of those using medication experienced complete relief (29.5% and 37.4%, respectively), while 10.6% reported mild relief (Table 2).

**Table 2:** Distribution of clinical data of headache by smartphone use level, Taif City, Saudi Arabia P: Pearson X2 test; $: exact probability test; * P < 0.05 (significant)

Headache clinical data	Total		Smartphone use level				P-value
			Low smartphone users		High smartphone users		
	No	%	No	%	No	%	
Headache							.025*
Migraine	86	21.3%	32	25.0%	54	19.6%	
Tension headache	33	8.2%	10	7.8%	23	8.4%	
Cluster headache	8	2.0%	6	4.7%	2	.7%	
Nonspecific type of chronic headache	38	9.4%	16	12.5%	22	8.0%	
Undiagnosed chronic headache	109	27.0%	31	24.2%	78	28.4%	
No chronic headache	129	32.0%	33	25.8%	96	34.9%	
Severity level (n=274)							.028*
Mild	28	10.2%	11	11.6%	17	9.5%	
Moderate	134	48.9%	36	37.9%	98	54.7%	
Severe	112	40.9%	48	50.5%	64	35.8%	
The change in your headache symptoms over the last year (n=274)							.108^$^
Improved	104	38.0%	39	41.1%	65	36.3%	
Stable	130	47.4%	48	50.5%	82	45.8%	
Worsened	40	14.6%	8	8.4%	32	17.9%	
How many pills do you take to treat acute headaches per month? (n=274)							.001*
0-5	166	60.6%	45	47.4%	121	67.6%	
5-10	56	20.4%	21	22.1%	35	19.6%	
> 10	52	19.0%	29	30.5%	23	12.8%	
Do you use any medication as a prophylaxis? (n=274)							.129
Yes	58	21.2%	25	26.3%	33	18.4%	
No	216	78.8%	70	73.7%	146	81.6%	
The relief from headaches after medication (n=274)							.171
Mild	29	10.6%	14	14.7%	15	8.4%	
Moderate	150	54.7%	53	55.8%	97	54.2%	
Complete	95	34.7%	28	29.5%	67	37.4%	

A total of 97.1% of study participants reported experiencing severe pain during headache episodes, with the frequency ranging from rarely to always. Additionally, 94.1% mentioned that headaches regularly impede their ability to engage in daily activities, including household chores, school, or social activities. Furthermore, 91.5% expressed a desire to lie down during headache episodes, while 89.1% acknowledged that headaches often restrict their ability to concentrate on work or daily activities. Additionally, 88.7% revealed feeling frustrated or irritated owing to headaches, and 87.2% frequently experienced fatigue, impacting their ability to perform work or daily activities (Table 3).

**Table 3:** Impact of headache among study participants in Taif City, Saudi Arabia n=274

Impact of headache	Never		Rarely		Sometimes		Very often		Always	
	No	%	No	%	No	%	No	%	No	%
When you have headaches, how often is the pain severe?	8	2.9%	24	8.8%	103	37.6%	83	30.3%	56	20.4%
How often do headaches limit your ability to do usual daily activities including household work, school, or social activities?	16	5.8%	42	15.3%	90	32.8%	72	26.3%	54	19.7%
When you have a headache, how often do you wish you could lie down?	23	8.4%	28	10.2%	60	21.9%	73	26.6%	90	32.8%
In the past four weeks, how often have you felt too tired to do work or daily activities because of your headaches?	35	12.8%	39	14.2%	89	32.5%	71	25.9%	40	14.6%
In the past four weeks, how often have you felt fed up or irritated because of your headaches?	31	11.3%	47	17.2%	79	28.8%	63	23.0%	54	19.7%
In the past four weeks, how often did headaches limit your ability to concentrate on work or daily activities?	30	10.9%	44	16.1%	85	31.0%	75	27.4%	40	14.6%

Among the patients with primary headaches, 64.6% (177 individuals) reported a significant effect on their daily lives, with 12.8% experiencing a substantial impact and 9.1% indicating little to no effect from headaches.

In the low smartphone user group, 67.4% reported a severe impact of headaches on their daily lives, slightly higher than the 63.1% reported by high smartphone users. Meanwhile, 8.4% of low users experienced little impact, compared to 9.5% of high users, and this difference did not reach statistical significance (P =.189) (Table 4).

**Table 4:** The relation between the level of smartphone use and headache impact on patient daily life P: Pearson X2 test

Headache impact	Smartphone use level				P-value
	Low smartphone users		High smartphone users		
	No	%	No	%	
Little impact	8	8.4%	17	9.5%	.189
Some impact	16	16.8%	21	11.7%	
Substantial impact	7	7.4%	28	15.6%	
Severe impact	64	67.4%	113	63.1%	

Migraine attacks resulted in a severe impact in 72.1% of cases, compared to 50% of cluster headache attacks (P =.042). Additionally, 35.7% of patients experiencing mild headaches reported severe impact, while 79.5% of those with severe headaches reported the same (P =.001). Similarly, 88.5% of patients consuming more than ten pills per month experienced a severe impact from headaches, compared to 56% of those taking zero to five pills (P =.003) (Table 5).

**Table 5:** The relation between headache clinical data and its impact on daily life activities P: Pearson X2 test; $: exact probability test; * P < 0.05 (significant)

Headache data	Headache impact (HIT-6)								P-value
	Little impact		Some impact		Substantial impact		Severe impact		
	No	%	No	%	No	%	No	%	
Headache									.042*^$^
Migraine	8	9.3%	7	8.1%	9	10.5%	62	72.1%	
Tension headache	6	18.2%	2	6.1%	7	21.2%	18	54.5%	
Cluster headache	2	25.0%	0	0.0%	2	25.0%	4	50.0%	
Nonspecific type of chronic headache	3	7.9%	5	13.2%	3	7.9%	27	71.1%	
Undiagnosed chronic headache	6	5.5%	23	21.1%	14	12.8%	66	60.6%	
Severity level									.001*^$^
Mild	3	10.7%	8	28.6%	7	25.0%	10	35.7%	
Moderate	15	11.2%	19	14.2%	22	16.4%	78	58.2%	
Severe	7	6.3%	10	8.9%	6	5.4%	89	79.5%	
Do you use any medication as a prophylaxis?									.227
Yes	3	5.2%	5	8.6%	6	10.3%	44	75.9%	
No	22	10.2%	32	14.8%	29	13.4%	133	61.6%	
The relief from headaches after medication									.081^$^
Mild	4	13.8%	2	6.9%	3	10.3%	20	69.0%	
Moderate	9	6.0%	18	12.0%	16	10.7%	107	71.3%	
Complete	12	12.6%	17	17.9%	16	16.8%	50	52.6%	
How many pills do you take to treat acute headaches per month									.003*
0-5	20	12.0%	26	15.7%	27	16.3%	93	56.0%	
5-10	4	7.1%	9	16.1%	5	8.9%	38	67.9%	
> 10	1	1.9%	2	3.8%	3	5.8%	46	88.5%	

## Discussion

The rapid improvement of technology has significantly transformed electronic media consumption patterns, especially with the widespread availability of smartphones. These devices have seamlessly integrated into our personal, social, and professional lives. Excessive mobile phone usage has been associated with various medical and psychological conditions. Thus, this study aimed to elucidate the intricate relationship between smartphone use and primary headaches. Additionally, its goal was to discern the types and pain severity levels experienced by patients with primary headaches. Furthermore, the study aimed to investigate variations in the need for acute medications and prophylactics.

Our study did not identify a significant correlation between excessive smartphone usage and primary headaches. The higher representation of female participants in both groups aligns with other studies [17], possibly attributed to their greater willingness to participate in surveys. In our demographic, most participants were recent university graduates, with over half falling into the high smartphone user category. This prevalence among working individuals may be attributed to job requirements involving phone usage. Interestingly, a study conducted in Turkey demonstrated a relationship between smartphone usage and headaches [12]. This discrepancy could stem from our sample comprising the general population, while their focus was explicitly on university students.

The predominant type of headache reported in both groups was undiagnosed chronic headache, in contrast to a study conducted in India, where migraines were identified as the most prevalent type of headache [10]. This disparity could be associated with variations in people's awareness of the importance of seeking medical attention for chronic headaches.

When assessing the severity of headaches among high and low smartphone users, our findings revealed that approximately half of the participants experienced moderate headaches, with more than half being high smartphone users. Surprisingly, participants with severe headaches tended to be low smartphone users, potentially indicating reduced smartphone usage among individuals dealing with more severe headache symptoms. A study from Pakistan reported an increase in pain intensity among high smartphone users with migraine headaches [11]. However, this difference may stem from our focus on different types of primary headaches. Furthermore, a notable observation was that the majority of high smartphone users used zero to five pills per month, while those using more than ten pills were predominantly low smartphone users.

In pursuit of our secondary objectives, we aimed to assess the severity of each primary headache type. Our analysis revealed that migraine was the headache type with the highest severity and intensity of symptoms, encompassing the majority of participants diagnosed with migraine. More than a quarter of these individuals reported experiencing little to substantial impact from the symptoms. In comparison, patients with cluster headaches and tension headaches exhibited a different pattern, with half to just over half, respectively, reporting severe impact. Less than half of tension headache patients reported little to substantial impact. In the case of cluster headache patients, approximately half of them denied experiencing severe impact.

We also studied the use of acute medications and prophylactic treatment. Our data revealed that most of the participants reported moderate consumption, relying on zero to five pills per month to alleviate their headaches. Conversely, a smaller yet significant portion of the participants relied on a higher dosage, exceeding ten pills per month. Notably, only a limited proportion of participants employed prophylactic treatment, suggesting a potential link between its underutilization and an increase in the frequency and severity of headaches, consequently leading to higher usage of acute medication. Among those resorting to medication, a substantial fraction experienced moderate relief from their headaches, while a smaller group reported mild relief. In contrast, a study in India found a higher frequency of using medications with a poor response to analgesics [10]. These findings emphasize the importance of tailoring treatment plans to individual needs, considering the frequency and type of drugs used, and weighing the potential benefits of prophylactic measures.

To our knowledge, this is the first study examining the correlation between smartphone use and primary headaches in Taif, Saudi Arabia. However, it is essential to acknowledge certain limitations of our study. The retrospective nature of the study and the lack of clinical evaluation to confirm headache diagnoses rely on participants' perceptions of headaches. Furthermore, our survey did not include specific details about the types of medications. Last but not least, more denials of participation in the survey from male patients may lead to some bias.

## Conclusions

This cross-sectional study revealed an absence of a correlation between smartphone use and the impact of headaches. Notably, a considerable portion of patients experiencing chronic headaches lacked a specific diagnosis, potentially attributed to a preference for analgesics over seeking a comprehensive diagnosis. Initiating public awareness campaigns regarding various types of headaches is imperative to enhance diagnosis and treatment for such patients. Future studies should employ measurable parameters such as call duration or screen time instead of relying solely on self-reported smartphone usage to assess their potential association more accurately.
